# Measures of subclinical cardiac dysfunction and increased filling pressures associate with pulmonary arterial pressure in the general population: results from the population-based Rotterdam Study

**DOI:** 10.1007/s10654-017-0341-0

**Published:** 2017-12-13

**Authors:** Ryan J. Billar, Maarten J. G. Leening, Daphne Merkus, Guy G. O. Brusselle, Albert Hofman, Bruno H. Ch. Stricker, H. Ardeschir Ghofrani, Oscar H. Franco, Henning Gall, Janine F. Felix

**Affiliations:** 1 Department of Epidemiology, Erasmus MC - University Medical Center Rotterdam, P.O. Box 2040, 3000 CA Rotterdam, The Netherlands; 2 Department of Cardiology, Erasmus MC - University Medical Center Rotterdam, Rotterdam, The Netherlands; 3 Department of Epidemiology, Harvard T.H. Chan School of Public Health, Boston, MA USA; 4 Department of Respiratory Medicine, Ghent University Hospital, Ghent, Belgium; 5 Department of Internal Medicine, Erasmus MC - University Medical Center Rotterdam, Rotterdam, The Netherlands; 6 Inspectorate for Health Care, Utrecht, The Netherlands; 7 Medizinische Klinik II, University of Giessen and Marburg Lung Center (UGMLC) - Member of the German Center for Lung Research (DZL), Giessen, Germany

**Keywords:** Echocardiography, Pulmonary artery systolic pressure, Pulmonary hypertension, Heart failure, Epidemiology, Population-based

## Abstract

Pulmonary hypertension is associated with increased mortality and morbidity in the elderly population. Heart failure is a common cause of pulmonary hypertension. Yet, the relation between left heart parameters reflective of subclinical cardiac dysfunction and increased filling pressures, and pulmonary arterial pressures in the elderly population remains elusive. Within the population-based Rotterdam Study, 2592 unselected participants with a mean age of 72.6 years (61.4% women) had complete echocardiography data available. We studied the cross-sectional associations of left heart structure and systolic and diastolic function with echocardiographically measured pulmonary artery systolic pressure. Mean pulmonary artery systolic pressure was 25.4 mmHg. After multivariable-adjustment measures of both structure and function were independently associated with pulmonary artery systolic pressure: E/A ratio [0.63 mmHg (95% CI 0.35–0.91) per 1-SD increase], left atrial diameter [0.79 mmHg (0.50–1.09) per 1-SD increase], E/E′ ratio [1.27 mmHg (0.92–1.61) per 1-SD increase], left ventricular volume [0.62 mmHg (0.25–0.98) per 1-SD increase], fractional shortening [0.45 mmHg (0.17–0.74) per 1-SD increase], aortic root diameter [− 0.43 mmHg (− 0.72 to − 0.14) per 1-SD increase], mitral valve deceleration time [− 0.31 mmHg (− 0.57 to − 0.05) per 1-SD increase], and E′ [1.04 mmHg (0.66–1.42) per 1-SD increase]. Results did not materially differ when restricting the analyses to participants free of symptoms of shortness of breath. Structural and functional echocardiographic parameters of subclinical cardiac dysfunction and increased filling pressures are associated with pulmonary arterial pressures in the unselected general ageing population.

## Introduction

Pulmonary hypertension can occur idiopathically or, most frequently, in association with a number of common clinical conditions, including heart failure and chronic obstructive pulmonary disease [1, 2]. Pulmonary hypertension represents the extreme form of increased pulmonary artery systolic pressure (PASP). Both pulmonary hypertension and increased PASP within its normal range are associated with significant morbidity and mortality, both in patients with underlying disorders and in the general population [1–3]. However, increased PASP often remains undetected until it is advanced, likely due to its nonspecific symptoms, including shortness of breath and fatigue [1–4].

Heart failure is a common cause of pulmonary hypertension. Underlying left ventricular failure represents the clinical phenotype on the extreme end of a continuum of left heart function. Yet, data on the relation between left heart parameters reflective of subclinical cardiac dysfunction and increased filling pressures, and pulmonary arterial pressures are scarce, especially in the elderly population at risk for heart failure [2, 5, 6]. Therefore, we aimed to determine the associations of echocardiographically measured left heart parameters across the full spectrum with PASP in the general ageing population.

## Methods

### Study population and setting

This study was conducted within the Rotterdam Study, an ongoing prospective population-based cohort study, started in 1990 in Rotterdam, the Netherlands. The rationale and design of the Rotterdam Study have been described in detail elsewhere [7, 8]. In short, the Rotterdam Study started in 1990–1993 (RS-I, 7983 participants of 55 years and older). Two additional subcohorts (RS-II, 3011 participants of 55 years and older, and RS-III, 3932 participants of 45 years and older) were recruited in 2000–2001 and 2006–2008, respectively. Every 3–4 years, participants undergo a home interview and clinical examinations at the Rotterdam Study research center. Data on clinical diagnoses of comorbidities, such as heart failure, are collected continuously throughout follow-up [9].

**Fig. 1:**
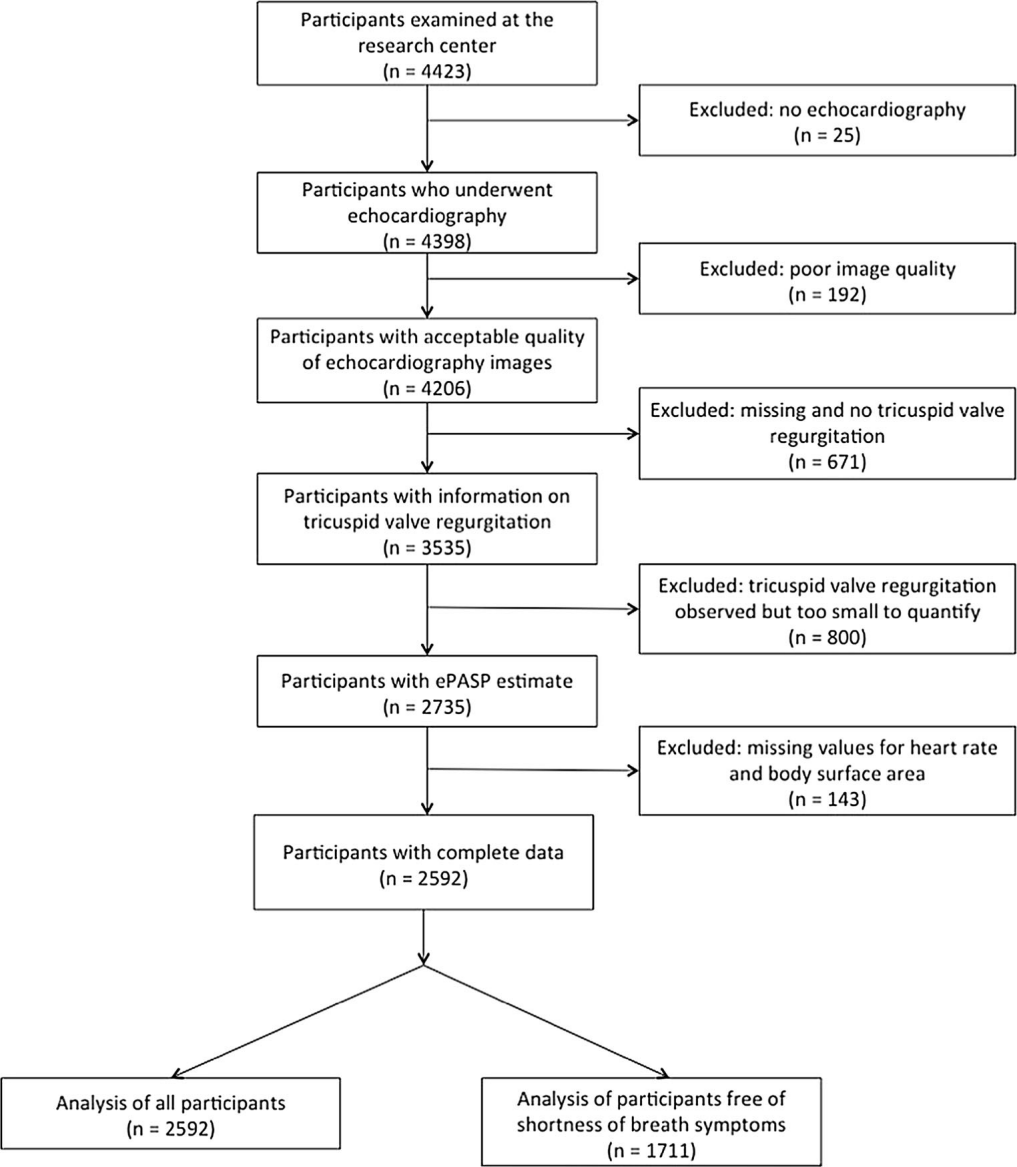
Flow chart of study participants

All 4423 participants who visited the research center during the fifth examination of RS-I (2009–2011), the third examination of RS-II (2011–2012) and the second examination of RS-III (2012) were eligible for this analysis. We excluded participants who did not undergo echocardiography (mostly due to logistic reasons), those with poor quality of echocardiography, and those in whom tricuspid valve regurgitation values were too small to measure, making it impossible to calculate echocardiographically measured pulmonary artery systolic pressure (ePASP). We also excluded participants without measurements of resting heart rate or body surface area, which were used as key co-variables. Our final analyses included 2592 participants (Fig. 1).

### Echocardiography

Resting transthoracic 2-dimensional echocardiography was performed by experienced echocardiographers, using an identical standardized protocol for all participants and using a commercially available ultrasonography system (Vivid I, GE Healthcare, Little Chalfont, UK), with a 2.5 MHz transducer. The protocol included 2-dimensional scanning in the parasternal long and short axis views, the apical and subcostal views. In addition, 2-dimension guided M-mode measurements of left ventricle and aortic root were obtained by scanning in the parasternal long axis view. Tissue Doppler imaging (TDI) was done in the apical 4-chamber view. All images were digitally stored and assessed offline by the echocardiographers.

Left-sided measurements were aortic root diameter (AoD), left atrial diameter (LAD), left ventricular end diastolic diameter (LVEDD), left ventricular end systolic diameter (LVESD), interventricular septum thickness (IVST), and left ventricular posterior wall thickness (LVPWT). Left ventricular mass (LVmass) in grams was calculated according to the formula by Devereux and colleagues as 0.8 * {1.04 * ([LVEDD + IVST + LVPWT]^3^ − LVEDD^3^)} + 0.6 [10]. Left ventricular volume (LVvol) in milliliters was calculated according to the formula by Teichholz and colleagues as 7 * LVEDD^3^/(2.4 + LVEDD) [11]. Left ventricular fractional shortening (LVFS) was calculated as (LVEDD-LVESD)/LVEDD * 100% [12].

Pulsed wave Doppler in the apical 4-chamber view was used to measure the early transmitral ventricular diastolic filling velocity (E wave) and late diastolic filling velocity (A wave) during 3 cardiac cycles. TDI was used to measure the early diastolic longitudinal filling velocity of the septal mitral annulus (E′) during 3 cardiac cycles. The means of the E wave, A wave and E′ over the 3 cardiac cycles were calculated and used to calculate E/A ratio and E/E′ ratio. Mitral valve deceleration time was measured as the time between the peak E-top wave and the upper deceleration slope extrapolated to the zero baseline using a Continuous Wave Doppler.

ePASP was calculated as the sum of the estimated right atrial pressure (RAP, based on inferior vena cava diameter and forced inspiratory collapse) and the pressure gradient over the tricuspid valve. RAP was estimated according to the algorithm recommended by the ASE/EAE/CSE [13]: if the inferior vena cava diameter was ≤ 21 mm and its forced inspiratory collapse (“sniff test”) was > 50%, RAP was considered to be 3 mmHg (n = 1939); if the diameter was > 21 mm and the collapse < 50%, RAP was considered as 15 mmHg (n = 41); in intermediate cases, a value of 8 mmHg was assigned (n = 380). Tricuspid regurgitation peak velocity (TRV) was measured using Continuous Wave Doppler. The tricuspid pressure gradient was estimated using the simplified Bernoulli formula as 4 * TRV^2^ [13]. Thus, ePASP was estimated as 4 * TRV^2^ + RAP. All 232 participants with measured TRV values but missing RAP were assigned a conservative RAP of 3 mmHg.

### Shortness of breath

Shortness of breath was assessed during the home interview, which included the following question: “Do you ever have shortness of breath?”. If the answer was yes, the participant was asked “Do you have shortness of breath at rest?”. If the answer was no, the participant was asked “Do you have shortness of breath when walking on a flat surface?”. If the answer was no, the participant was asked “Do you have shortness of breath when climbing stairs?”. If participants answered no to the first question, or if they answered yes to the first question but no to the other 3, they were classified as being free of significant shortness of breath. This is in line with the New York Heart Association (NYHA) validity class I [14].

### Co-variables

Information routinely collected in every follow-up round of the Rotterdam Study includes anthropometrics, cardiovascular risk factors, and medication use. Body surface area in m^2^ was calculated as 0.007184 * (height in cm)^0.725^ * (weight in kg)^0.425^ [15]. Resting heart rate was measured during blood pressure measurement using an Omron M7 pulse blood pressure monitoring device. Heart rate was measured twice with a time-interval of 5 min between both measurements. The mean of the 2 measurements was used in the analyses. Medication use was coded according to the Anatomical Therapeutical Chemical coding system of the World Health Organization [16].

### Statistical analyses

We fitted multivariable linear regression models to study the cross-sectional association of ePASP as the dependent variable with each of the following independent echocardiographic variables separately: LAD, AoD, mitral valve deceleration time, IVST, LVPWT, LVmass, LVvol, LVEDD, left ventricular fractional shortening, E/A ratio, and E/E′ ratio. All models were adjusted for sex, age, resting heart rate, and body surface area.

We also fitted a larger multivariable-adjusted linear regression model with ePASP as the dependent variable and LAD, AoD, mitral valve deceleration time, LV mass, LVvol, left ventricular fractional shortening, E/A ratio, E/E′ ratio, and E′, as well as sex, age, resting heart rate, and body surface area. LVvol and LVmass are calculated based on the measured LVEDD, IVST, and LVPWT. Therefore, we chose to use the clinically more relevant measures of LVvol and LVmass in the larger model instead of the underlying measurements.

We also analyzed the effect of clinical categories of E/A ratio and E/E′ ratio instead of using the continuous measures. E/A ratio was divided into three categories: ≤ 0.75, > 0.75 and < 1.50, and ≥ 1.50. We divided E/E′ ratio also into three categories: ≤ 8, > 8 and < 15, and ≥ 15 [17, 18]. To study whether our results were driven by symptomatic individuals we repeated the analysis in participants free of significant shortness of breath. We tested for potential interaction between the echocardiographic variables and sex by adding multiplicative interaction terms to the regression models. The interaction terms were not significant after Bonferroni correction for multiple testing (*P* value cutoff was 0.00625), thus we did not stratify by sex.

Effect estimates are presented as βs (both per unit increase and per 1-standard deviation (SD) increase in the left-sided measurement) with 95% confidence intervals (95% CIs). We considered *P* values of < 0.05 statistically significant. All analyses were done using IBM SPSS Statistics version 21.0 (IBM Corp., Somers, NY, USA).

## Results

Participant characteristics and echocardiographic measurements are presented in Table 1. The mean age was 72.6 (SD 8.7) years and 61.4% of the participants were women. The mean ePASP was 25.4 (SD 6.7) mmHg. A total of 2.8% of participants included in this analysis had an ePASP of > 40 mmHg or, in absence of an RAP measurement, a TRV > 3.0, which indicates pulmonary hypertension based on echocardiography.

**Table 1:** Demographic, clinical and echocardiographic characteristics of included participants, n = 2592

	Mean (SD) or n (%)
Age (years)	72.6 (8.7)
Women	1591 (61.4%)
Height (cm)	167 (9)
Weight (kg)	75.0 (13.4)
Body surface area (m^2^)	1.83 (0.19)
Body mass index (kg/m^2^)	26.9 (4.0)
Systolic blood pressure (mmHg)	147 (22)
Diastolic blood pressure (mmHg)	84 (11)
Resting heart rate (bpm)	68 (11)
History of chronic obstructive pulmonary disease	267 (10.3%)
History of atrial fibrillation	163 (6.9%)
Shortness of breath^a^	
Validity class I	1711 (73.2%)
Validity class II	220 (9.4%)
Validity class III	271 (11.6%)
Validity class VI	135 (5.8%)
Smoking	
Never	944 (36.4%)
Former	1377 (53.2%)
Current	269 (10.4%)
History of diabetes mellitus	243 (9.6%)
Blood pressure-lowering drugs	1131 (55.9%)
Diuretics	465 (23.0%)
β-blockers	639 (31.6%)
Calcium channel blockers	232 (11.5%)
RAAS inhibitors	640 (31.7%)
Other	15 (0.7%)
Cholesterol-lowering drugs	617 (30.5%)
Echocardiography	
Pulmonary arterial systolic pressure (mmHg)	25.4 (6.7)
Pulmonary arterial systolic pressure > 40 mmHg	73 (2.8%)
Left ventricular mass (gram)	130 (38)
Left ventricular volume (mL)	126 (31)
Interventricular septum thickness (mm)	8 (2)
Left ventricular posterior wall thickness (mm)	7 (1)
Diameter of left atrium (mm)	42 (6)
Left ventricular fractional shortening (%)	42 (6)
Left ventricular end-diastolic dimension (mm)	51 (5)
Diameter of the aortic root (mm)	34 (4)
E/A ratio	0.93 (0.35)
≤ 0.75	741 (29.9%)
> 0.75 and < 1.50	1636 (66.1%)
≥ 1.50	98 (4.0%)
E/E′ ratio	11.0 (4.8)
≤ 8	620 (23.1%)
> 8 and < 15	1714 (63.8%)
≥ 15	352 (13.1%)
E′ (m/s)	0.07 (0.02)
Mitral valve inflow deceleration-time (ms)	204 (42)

Missing values for data on traditional cardiovascular risk factors ranged from 0 to 2.8%

Missing values for data on left heart echocardiographic parameters ranged from 0.2 to 1.7%, with exception of E/A ratio with 5.2% (mainly due to atrial fibrillation)

*bpm* beats per minute, *E/A ratio* E wave divided by A wave, *E/E′ ratio* E wave divided by E′, *HDL* high density lipid protein, *RAAS* renin–angiotensin–aldosterone system

^a^Validity classes according to the New York Heart Association (NYHA) [14], data available in 90.2% of the participants

**Fig. 2:**
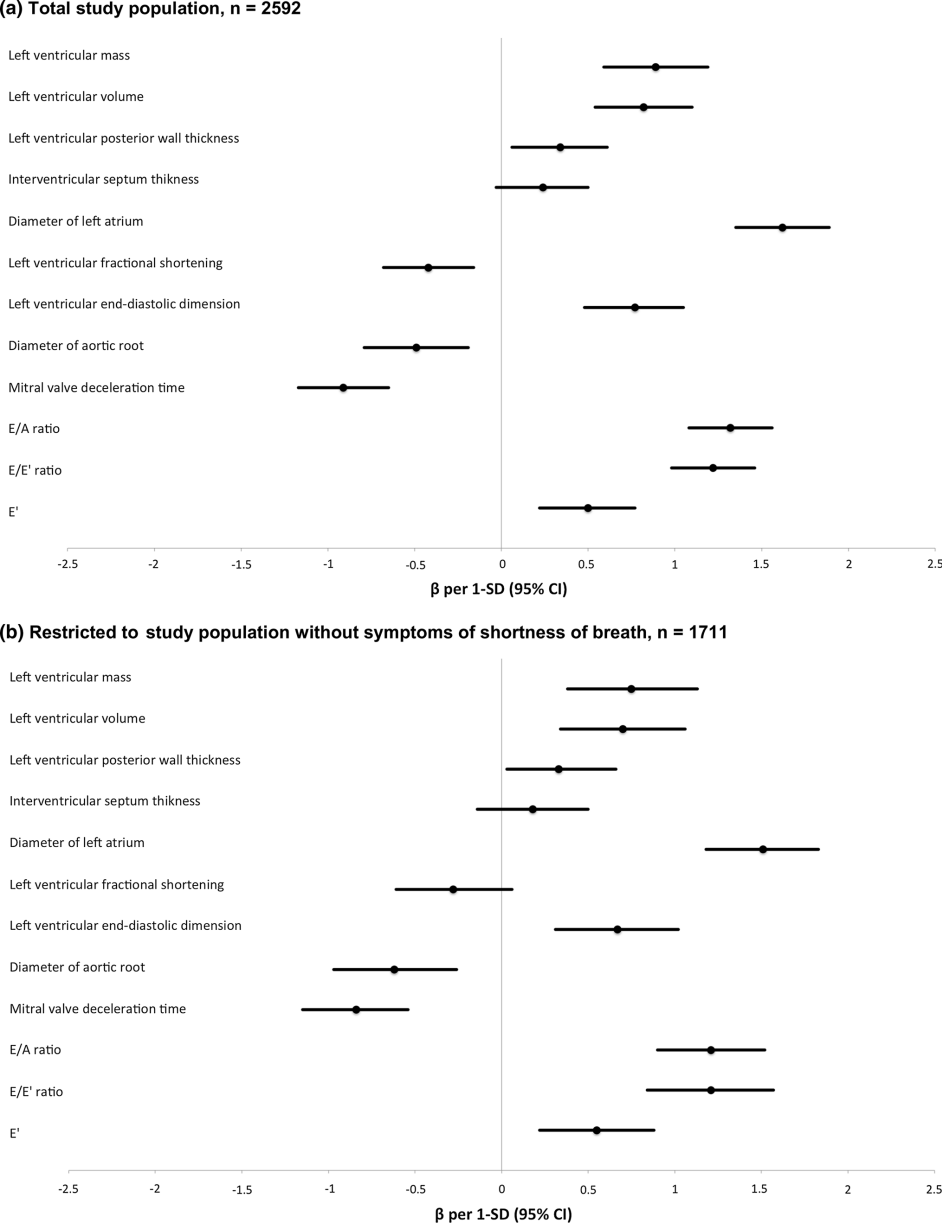
Associations of individual left heart echocardiographic parameters with ePASP. **a** Total study population, n = 2592, **b** restricted to study population without symptoms of shortness of breath, n = 1711. Adjusted for age, sex, resting heart rate, and body surface area

In the basic model, all parameters studied were significantly related to ePASP, except for IVST. Table 2 and Fig. 2a show the results per unit and per 1-SD increase, respectively. The variables with the strongest associations with ePASP were LAD, E/A ratio, E/E′ ratio, and LVvol.

**Table 2:** Associations of individual left heart echocardiographic parameters with ePASP

	Full study population, n = 2592		Restricted to free of shortness of breath, n = 1711	
	Difference (95% CI) in ePASP	*P* value	Difference (95% CI) in ePASP	*P* value
Left ventricular mass (per 10 grams)	0.23 (0.15 to 0.30)	< 0.001	0.19 (0.10 to 0.29)	< 0.001
Left ventricular volume (per 10 mL)	0.26 (0.27 to 0.35)	< 0.001	0.22 (0.11 to 0.34)	< 0.001
Left ventricular posterior wall thickness (mm)	0.28 (0.05 to 0.51)	0.016	0.28 (0.00 to 0.55)	0.048
Interventricular septum thickness (per 1 mm)	0.15 (− 0.02 to 0.32)	0.078	0.12 (− 0.09 to 0.32)	0.264
Diameter of left atrium (per 1 mm)	0.27 (0.22 to 0.31)	< 0.001	0.25 (0.19 to 0.30)	< 0.001
Left ventricular fractional shortening (per 1%)	− 0.07 (− 0.12 to − 0.03)	0.002	− 0.05 (− 0.11 to 0.01)	0.102
Left ventricular end-diastolic dimension (per 1 mm)	0.14 (0.09 to 0.20)	< 0.001	0.13 (0.06 to 0.19)	< 0.001
Diameter of the aortic root (per 1 mm)	− 0.12 (− 0.20 to − 0.05)	0.001	− 0.15 (− 0.24 to − 0.07)	0.001
Mitral valve inflow deceleration-time (per 10 ms)	− 0.22 (− 0.28 to − 0.15)	< 0.001	− 0.20 (− 0.27 to − 0.13)	< 0.001
E/A ratio	3.94 (3.23 to 4.65)	< 0.001	3.60 (2.67 to 4.53)	< 0.001
≤ 0.75	0.00 (reference)		0.00 (reference)	
> 0.75 and < 1.50	1.55 (0.98 to 2.12)	< 0.001	1.47 (0.79 to 2.15)	< 0.001
≥ 1.50	6.24 (4.94 to 7.54)	< 0.001	5.69 (4.05 to 7.32)	< 0.001
E/E′ ratio	0.27 (0.22 to 0.32)	< 0.001	0.27 (0.19 to 0.35)	< 0.001
≤ 8	0.00 (reference)		0.00 (reference)	
> 8 and < 15	1.08 (0.46 to 1.70)	0.001	1.09 (0.36 to 1.83)	0.003
≥ 15	3.36 (2.44 to 4.28)	< 0.001	3.36 (2.24 to 4.48)	< 0.001
E′ (per 0.1 m/s)	2.51 (1.12 to 3.89)	< 0.001	2.77 (1.11 to 4.42)	0.001

Adjusted for age, sex, resting heart rate, and body surface area

*ePASP* echocardiographic pulmonary artery systolic pressure, *SD* standard deviation, *CI* confidence interval, *E/A ratio* E wave divided by A wave, *E/E′ ratio* E wave divided by E′

**Fig. 3:**
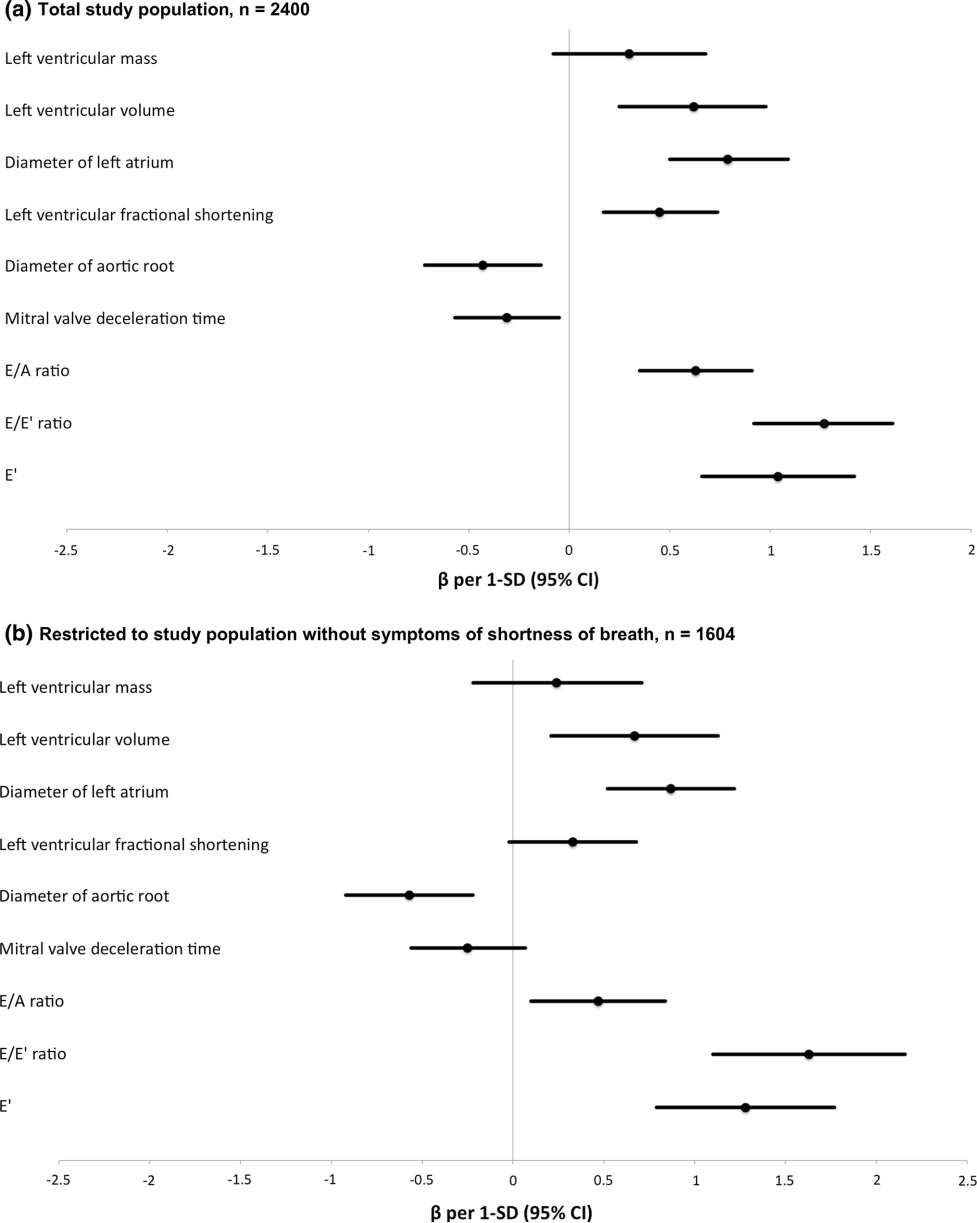
Multivariable-adjusted associations of left heart echocardiographic parameters with ePASP. **a** Total study population, n = 2400, **b** restricted to study population without symptoms of shortness of breath, n = 1604. Adjusted for age, sex, heart rate, body surface area, and all other left heart echocardiographic parameters in this figure

In the larger multivariable-adjusted model, ePASP was associated with E/A ratio [0.63 mmHg (95% CI 0.35–0.91) per 1-SD increase], LAD [0.79 mmHg (0.50–1.09) per 1-SD increase], E/E′ ratio [1.27 mmHg (0.92–1.61) per 1-SD increase], and LVvol [0.62 mmHg (0.25–0.98) per 1-SD increase], LVFS [0.45 mmHg (0.17–0.74) per 1-SD increase], AoD [− 0.43 mmHg (− 0.72 to − 0.14) per 1-SD increase], E′ [1.04 mmHg (0.66–1.42) per 1-SD increase], and mitral valve deceleration time [− 0.31 mmHg (− 0.57 to − 0.05) per 1-SD increase]. Table 2 and Fig. 3a show the results per 1-unit and per 1-SD increase in measurements, respectively. The directions of effect for all associations remained the same as in the basic models (Table 2 and Fig. 2a), except for LVFS, which was associated with a lower ePASP in the basic model and with a higher ePASP in the large model.

When restricting the analysis to the 1604 participants free of shortness of breath in the larger model, point estimates were similar to those obtained in the full study population. Table 3 and Fig. 3a show the results per 1-unit and per 1-SD increase in the left heart measurements, respectively. In the larger model, all echocardiographic parameters remained significantly associated with ePASP, except LVmass, fractional shortening and mitral valve deceleration time. In this model the strongest associations with ePASP were found for LAD, LVvol, E/A ratio, E/E′ ratio, and E′. Table 3 and Fig. 3b show the results per 1-unit and per 1-SD increase in the left heart measurements, respectively.

**Table 3:** Multivariable-adjusted associations of left heart echocardiographic parameters and ePASP

	Full study population, n = 2400		Restricted to free of shortness of breath, n = 1604	
	Difference (95% CI) in ePASP	*P* value	Difference (95% CI) in ePASP	*P* value
Left ventricular mass (per 10 grams)	0.08 (− 0.02 to 0.17)	0.117	0.06 (− 0.06 to 0.18)	0.202
Diameter of left atrium (per 1 mm)	0.13 (0.08 to 0.18)	< 0.001	0.14 (0.09 to 0.20)	< 0.001
Left ventricular fractional shortening (per 1%)	0.08 (0.03 to 0.13)	0.002	0.06 (− 0.00 to 0.12)	0.067
Left ventricular volume (per 10 mL)	0.19 (0.08 to 0.31)	0.001	0.21 (0.07 to 0.36)	0.004
Diameter of the aortic root (per 1 mm)	− 0.11 (− 0.18 to − 0.04)	0.003	− 0.14 (− 0.23 to − 0.06)	0.001
Mitral valve inflow deceleration-time (per 10 ms)	− 0.07 (− 0.14 to − 0.01)	0.021	− 0.06 (− 0.13 to 0.02)	0.124
E/A ratio	1.88 (1.05 to 2.71)	< 0.001	1.39 (0.29 to 2.49)	0.013
≤ 0.75	0.00 (reference)	NA	0.00 (reference)	NA
> 0.75 and < 1.50	0.40 (− 0.21 to 1.01)	0.197	0.36 (− 0.37 to 1.10)	0.329
≥ 1.50	2.90 (1.50 to 4.31)	< 0.001	2.48 (0.71 to 4.25)	0.006
E/E′ ratio	0.28 (0.20 to 0.36)	< 0.001	0.36 (0.24 to 0.48)	< 0.001
≤ 8	0.00 (reference)	NA	0.00 (reference)	NA
> 8 and < 15	1.05 (0.38 to 1.72)	0.002	1.52 (0.71 to 2.34)	< 0.001
≥ 15	2.84 (1.69 to 3.98)	< 0.001	3.82 (2.41 to 5.24)	< 0.001
E′ (per 0.1 m/sec)	5.23 (3.32 to 7.14)	< 0.001	6.47 (4.00 to 8.94)	< 0.001

Adjusted for age, sex, resting heart rate, body surface area, and all other continuous left heart echocardiographic parameters in this table

*ePASP* echocardiographic pulmonary artery systolic pressure, *SD* standard deviation, *CI* confidence interval, *E/A ratio* E wave divided by A wave, *E/E′ ratio* E wave divided by E′

## Discussion

In our study among older community-dwelling adults, structural and functional left heart parameters reflective of subclinical cardiac dysfunction and increased filling pressures were associated with ePASP. Most of the associations persisted when we restricted the analysis to participants free of shortness of breath, implying that these associations are not driven by individuals with overt heart failure.

Our findings corroborate with three recent other epidemiologic studies. The first included 6598 male recruits from the Israeli air force. The authors reported significant associations of, LAD, and LVEDD with ePASP [6]. Furthermore, LVmass was not significantly related to ePASP in the Israeli study, which is in line with our findings. In contrast to our findings, the authors observed no significant association of LVFS with ePASP. This difference might be explained by the differences in age and health status of the study participants. When we restricted our analysis to participants without shortness of breath, and hence to the healthier part of our population, left ventricular fractional shortening was also no longer significantly related to ePASP in our study. The second study was done in 1480 younger Italians (mean age 36 years) free of structural heart disease. Results were similar, with a significant association of left ventricular stroke volume and E/E′ ratio with ePASP and no significant association with left ventricular mass index [4]. The third study included 3282 middle-aged African-Americans (mean age 56 years) and evaluated echocardiographic correlates of pulmonary hypertension rather than the entire spectrum of pulmonary artery pressures [5]. This study reported a significant association for LAD and for LV ejection fraction < 50% with presence of pulmonary hypertension. These results are overall in line with ours, although the latter association with LV systolic function attenuated in our multivariable-adjusted analysis.

### Mechanisms

We hypothesized that left-sided echocardiographic parameters across the full spectrum would be associated with ePASP, as early alterations in left heart structure and function can herald increased filling pressures and manifest heart failure, which is a common cause of pulmonary hypertension. Remarkably, LVFS showed a change in the direction of effect after multivariable-adjustment. This warrants further research to elucidate the underlying mechanisms involved.

Also, we observed that a greater AoD was associated with a lower ePASP. A similar association between AoD and ePASP was reported in the Israeli study [6]. The Framingham Heart Study previously reported an association between higher arterial systolic blood pressure and aortic root size in a population-based setting [19]. Concomitant stiffening of both the aortic and pulmonary artery walls, reducing the compliance of both, might explain these findings. This reduced compliance would cause a higher pulse pressure in the aortic root and a higher pressure in the pulmonary artery, as well as a smaller aortic root diameter [19]. However, this hypothesis warrants further fundamental experimental research.

### Limitations

Some limitations of our study need to be addressed. First, right heart catheterization is the gold standard for pulmonary arterial pressure measurements, but due to its invasiveness, heart catheterization is not suitable for population-based studies. However, several studies have shown that ePASP correlates well with invasively measured pulmonary arterial pressure and that echocardiography is appropriate for measurement of PASP in this setting [20, 21]. Also, the largest differences between pulmonary pressure measured by heart catheterization and echocardiography arise in those with high pressures [22]. The vast majority of the included participants had pressure estimates in the normal range. Second, TRV measurements could not be assessed in 621 participants (Fig. 1 and Table 1), predominantly because of technical difficulties in adequately visualizing the tricuspid valve. These participants were on average older (mean age 74.7 years), had a higher BMI (mean BMI 32.0 kg/m^2^), had a slightly worse left ventricular systolic function (mean LVFS 40%), and were more likely to report shortness of breath (47.5%). Thus, these participants were overall less healthy. Another 850 participants had undergone echocardiography, but had no tricuspid regurgitation or too small to quantify indicating they had a very low ePASP. These missing values will have affected the distribution of the studied echocardiographic parameters in our sample and resulted in an overestimation of the average ePASP, but are unlikely to have affected the β-estimates since we found no indications for nonlinearity in the data. Third, our population was older (mean age 72.6 years) and predominantly white (96.5%). Our results should therefore be extrapolated with caution to younger populations or those of other ethnic origins. Last, we did not have data available on more advanced or novel echocardiographic parameters, such as volumetric tracings, tissue Doppler-derived systolic function, and measures of strain.

### Clinical perspectives

Our work should be considered preclinical as this is the first study in the community-dwelling ageing population to demonstrate that parameters of subclinical left heart dysfunction and increased filling pressures correlate with subtle increases in pulmonary pressures. It remains to be studied whether progression to overt symptomatic pulmonary hypertension could be halted or controlled by pharmacological treatment in persons with subclinical left heart disease [23–26].

### Conclusion

Structural and functional echocardiographic parameters of subclinical cardiac dysfunction and increased filling pressures are associated with pulmonary arterial pressures in the unselected general ageing population.
